# Regional vessels wrapping following pancreaticoduodenectomy reduces the risk of post-operative extra-luminal bleeding. A systematic review

**DOI:** 10.1016/j.amsu.2022.104618

**Published:** 2022-09-14

**Authors:** Hussameldin M. Nour, Dimitra V. Peristeri, Amiya Ahsan, Shehram Shafique, Prof Mansoor Khan, Muhammad S. Sajid

**Affiliations:** a Department of Digestive Disease and General Surgery, Royal Sussex County Hospital, UHSussex NHS Trust, Eastern Road Brighton BN2 5BE, UK; b Warwick Medical School, Medical School Building, Clifford Bridge Rd, Coventry, CV4 7HL, UK

**Keywords:** Pancreatic neoplasm, Pancreatoduodenectomy, Whipple, Wrapping, Post-pancreatoduodenectomy haemorrhage

## Abstract

**Background:**

Post-pancreatectomy bleeding is a potentially fatal complication which results from the erosion of the regional visceral arteries, mainly the hepatic artery and stump of the gastro-duodenal artery, caused by a leak or fistula from the pancreatic anastomosis. The objective of this article is to assess whether wrapping of regional vessels with omentum or falciform/teres ligament following pancreaticoduodenectomy reduces the risk of extra-luminal bleeding.

**Materials and method:**

Standard medical electronic databases were searched with the help of a local librarian and relevant published randomised controlled trials (RCT) and any type of comparative trial were shortlisted according to the inclusion criteria. The summated outcome of post-operative extra-luminal bleeding in patients undergoing pancreaticoduodenectomy was evaluated using the principles of meta-analysis on RevMan 5 statistical software.

**Result:**

Two RCTs and 5 retrospective studies on 4100 patients undergoing pancreaticoduodenectomy were found suitable for this meta-analysis. There were 1404 patients in the wrapping-group (WG) and 2696 patients in the no-wrapping group (NWG). In the random effects model analysis, the incidence of extra-luminal haemorrhage was statistically lower in WG [odds ratio 0.51, 95%, CI (0.31, 0.85), Z = 2.59, P = 0.01]. There was moderate heterogeneity between the studies; however it was not statistically significant.

**Conclusion:**

The wrapping of regional vessels (using omentum, falciform ligament or ligamentum teres) following pancreaticoduodenectomy seems to reduce the risk of post-operative extra-luminal bleeding. However, more RCTs of robust quality recruiting a greater number of patients are required to validate these findings as this study presents the combined data of two RCTs and 5 retrospective studies.

## Highlights

• Post pancreaticoduodenectomy haemorrhage can be mortality and morbidity increasing complication.
• The aim is to assess the effectiveness of regional vessels wrapping in decreasing post operative bleeding.
• Two RCTs and five retrospective studies were used to obtain the submitted data.
• Regional vessels wrapping was associated with reduced bleeding post pancreaticoduodenectomy.

## 1 Introduction

Pancreatoduodenectomy is one of the most advanced and complicated surgeries for pancreatic, ampullary and duodenal neoplastic lesions in gastroenterology. Among the most pronounced and commonly listed postoperative complications of pancreaticoduodenectomy are pancreatic and biliary fistula, delayed gastric emptying and abscess formation [1]. Pancreatic fistula, which have an incidence ranging between 20 and 30% [2,3], may directly expose skeletonized or divided vessels, especially the gastroduodenal artery stump, to active pancreatic juice, forming a region that may result in vessel erosion or even delayed post-pancreatectomy haemorrhage (PPH) [4]. PPH is a rare but potentially very serious complication. It can occur as an early complication within the first 24 h, usually due to technical failures of inadequate haemostasis and perioperative coagulopathy, or late complication after the first post-operative day, usually resulting from erosion of the regional visceral arteries, mainly the hepatic artery and stump of the gastroduodenal artery, due to a leak or fistula from the pancreatic anastomosis. The rate of erosion related PPH has been reported up to 3–9%, however, the associated mortality rate is as high as 40%, even at specialized high-volume centres [5,6]. Patients who develop PPH have prolonged hospital stays with one study finding a median length of stay of 23 days, including 3 days of intensive care unit stay and total hospital costs of up to €55,623 [7].

In recent decades the development of perioperative care, high-quality CT scans and interventional radiology involvement in managing postoperative bleeding have helped to reduce mortality in patients undergoing pancreaticoduodenectomy [8]. However, the rate of complications following pancreaticoduodenectomy is still considered high and the management of late PPH can be quite challenging. Therefore, various surgical techniques have been employed at the time of the index operation to reduce the risk of postoperative complications. These include covering the gastroduodenal artery stump and hepatic artery or pancreatojejunostomy anastomosis with omental flap, falciform ligament or ligament of Treitz [4,5,9,10]. In various other studies, using different methods of wrapping of the regional blood vessels has be shown to be effective in reducing PPH [4,5,[9], [10], [11], [12], [13]].

The aim of this study is to assess whether regional vessels wrapping with omentum or falciform/teres ligament following pancreaticoduodenectomy reduces the risk of post-operative extra-luminal bleeding.

## 2 Methods

### 2.1 Data sources and literature search technique

A literature search using standard electronic databases including MEDLINE, EMBASE and Cochrane Library was conducted to identify suitable articles for this meta-analysis. The MeSH search terms issued in the Medline library related to the target objective were used to hit upon the relevant randomised controlled and retrospective trials. In this search, there were no limits for language, gender or sample size and place of study origin was recorded. Boolean operators (AND, OR, NOT) were used to narrow and widen the search results. The titles from the search results were carefully inspected and found to be appropriate for potential inclusion or exclusion into the study. Furthermore, the references from chosen articles were studied as an additional search strategy to find extra trials.

### 2.2 The inclusion criteria

All the studies had to compare the risk of post-operative extra-luminal bleeding between wrapping the regional vessels with omentum or falciform/teres ligament and no-wrapping the regional vessels following pancreaticoduodenectomy, in order to be included in this meta-analysis.

### 2.3 Collection of the data

All reported data was obtained by two different reviewers on a predefined meta-analysis data extraction form. It was matched and found to be in reasonable inter-reviewer agreement. The extracted data consisted of a list of the authors, title of the published study, journal of publication, country and year of the publication, testing sample size (with sex differentiation if applicable), the number of patients in each group based on the wrapping of the regional blood vessels or not, treatment protocol for each intervention, postoperative bleeding, and duration of follow up. Following the completion of data extraction, a thorough discussion took place between the independent reviewers and, if any differences were found, a mutual agreement was established.

### 2.4 Evidence construction

For statistical analysis, the software package RevMan 5 (The Nordic Cochrane Centre, Copenhagen, Denmark) [14,15] provided by the Cochrane Collaboration was used. The risk ratio (RR) with a 95% confidence interval (CI) was used in order to present the summated outcome for binary data. The random-effects model [16,17] was used to calculate the combined outcomes. Heterogeneity among included studies was explored using the chi^2^ test, with significance set at p < 0.05, and was quantified [18] using the I^2^ test with a maximum value of 30% identifying low heterogeneity [18]. The Mantel-Haenszel method was used for the calculation of RR under the random effect model [19] analysis. In a sensitivity analysis, 0.5 was added to each cell frequency for trials in which no event occurred in either the treatment or control group, according to the method recommended by Deeks et al. [20]. If the standard deviation was not available, then it was calculated according to the guidelines provided by the Cochrane Collaboration [16]. This process involved assumptions that both groups had the same variance, which may not have been true, and variance was either estimated from the range or from the p-value. The estimate of the difference between both techniques was pooled, depending upon the effect weights in results determined by each trial estimate variance. A forest plot was used for the graphical display of the results. The square around the estimate stood for the accuracy of the estimation (sample size), and the horizontal line represented the 95% CI. The methodological quality of the included trials was initially assessed using the published guidelines of Jaddad et al., Chalmers et al. and Rangel et al. [[21], [22], [23]].

### 2.5 Endpoint

Post-operative extra-luminal bleeding or PPH following pancreaticoduodenectomy was examined as the primary endpoint in this meta-analysis comparing wrapping group (WG) versus no-wrapping group (NWG).

### 2.6 PRISMA 2020 statement compliance

The conduction of this research work, writing the manuscript and submission work is in accordance with the PRISMA criteria [24]. The AMSTAR 2 criteria to assess the quality of this systematic review was applied and was more than 95% satisfactory [25].

## 3 Results

**Fig. 1:**
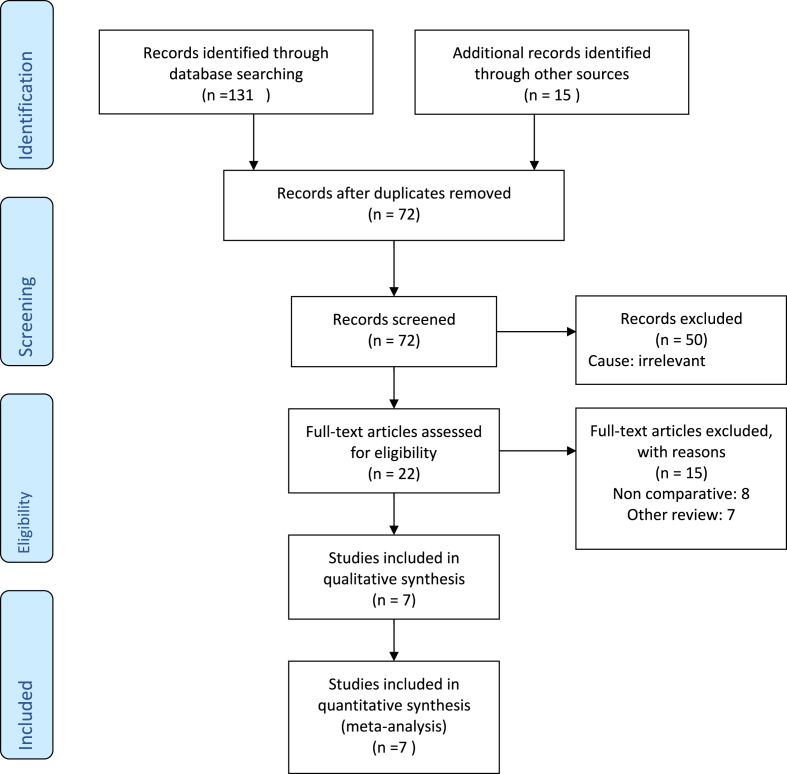
Prisma flow chart showing literature search outcomes.

The search of the standard medical electronic databases generated 72 studies after removing duplicated studies. The titles and abstracts of 72 studies were assessed, and 50 studies were considered irrelevant. The remaining 22 studies were further examined and only 7 studies were found to be eligible to be included in the systematic review (Fig. 1).

### 3.1 Qualities of studies and patients

Two RCTs [5,9] and five retrospective studies [4,[10], [11], [12], [13]] on 4100 patients fulfilled the inclusion criteria to conduct this meta-analysis based upon the principles provide by the Cochrane Collaboration. The PRIMA flow chart in trial search, trial deletion, trial selection and inclusion are given in Fig. 1. The included trials were conducted in Japan [[10], [11], [12]], Germany [5] and China [4]. The number of patients involved varied between the trials, ranging from 229 [10] to 2597 [12]. All the trials were conducted between 2012 [10] and 2022 [9]. Three studies [4,5,9] reported 90 days as the duration of follow up, whereas 60 days [11] and 2–3 weeks [10] were reported as follow up durations in two trials. In the remaining two trials [12,13] the follow up period was not clearly mentioned. The mean age of patients included in the trial ranged from 59.6 ± 12.3 [4] to 68(59–76) [9]. There was no discrimination for study selection in terms of gender, age, number of recruited patients or language of the published study. The main characteristics of the included studies are given in Table 1 and the treatment protocol adopted in each of the studies is given in Table 2.

**Table 1:** Characteristic of included studies.

Study	Year	Country	Study design	Number of patients	Age (Mean) years	Arms of the study
Matsuda [10]	2012	Japan	Retrospective	229	64.7 ± 10.8	Omental wrap versus no wrap
Meng [4]	2021	China	Retrospective	247	Wrap Group: 60.0 ± 13.1 non-Wrap group: 59.6 ± 12.3	Ligamentum teres wrap versus no wrap
Mussle [5]	2017	Germany	Randomized controlled trial	400	6 3 (53; 72)	Flaciform legmaent wrap versus no wrap
Okada [11]	2020	Japan	Retrospective	500	67.5 ± 12	Falciform ligament wrap versus no wrap
Tani [12]	2012	Japan	Retrospective	2597	67 (±10)	Omental wrap versus no wrap
Welsch [9]	2022	Germany	Randomized controlled trial.	445	68 (59–76)	Falciform ligament wrap versus no wrap
Xu [13]	2014	China	Retrospective	280	Wrap Group: 55.8 ± 10.0 non-Wrap group: 55.7 ± 10.8	Ligamentum teres wrap versus no wrap

**Table 2:** **Treatment protocol adopted in the included studies**.

Study	Wrap group	No wrap group	Follow up duration
Matsuda [10]	The omental flap was brought behind the pancreatoenteric anastomosis to cover the skeletonized hepatic arteries, superior mesenteric artery, splenic artery and portal vein, and fixed around the lesser omentum, the hepatic hilum and the jejunal limb to completely separate them of the pancreatoenteric anastomosis.	Same surgical technique without wrapping of the regional vessels	2–3 weeks
Meng [4]	The gastroduodenal artery stump (GDA) was exposed. The LTH was mobilized by dividing it around the GDA stump. The vessels between the ligament and liver parenchyma were ligated and divided. Using this method, we achieved a flap length of approximately 10 cm. The GDA stump was routinely fixed with 4–0 or 3–0 polypropylene sutures.	Same surgical technique without wrapping of the regional vessels	90 Days
Mussle [5]	After completion of the pancreatic, bile duct, or gastric/duodenal anastomosis, the prepared pedicled falciform ligament is carefully tunnelled below the common hepatic artery and wrapped around the GDA stump in a tension free fashion using only one turn. Fixation is then performed with two to three stitches using polydioxanone (PDS) 5–0.	Same surgical technique without wrapping of the regional vessels	90 days
Okada [11]	At laparotomy, the falciform ligament was cut at the point of its attachment to the abdominal wall. After the removal of pancreatoduodenal specimens, the gastroduodenal artery (GDA) stump and other major vessels (e.g., hepatic, splenic, and superior mesenteric artery), along with the preserved nerve plexus, were exposed adjacent to the pancreatic stump (We fixed the pedicled falciform ligament and retroperitoneal tissues circumferentially to separate the major vessels completely from the pancreatic anastomosis. We used 4–0 composition absorbent braid suture thread.	Same surgical technique without wrapping of the regional vessels	60 days
Tani [12]	Wrapping was performed at 2 locations: wrapping of vessels, including the common hepatic artery, proper hepatic artery, GDA stump, and portal vein and wrapping of pancreatic enterostomy.	Same surgical technique without wrapping of the regional vessels	Early intra-abdominal haemorrhage: 3 days Late intra-abdominal haemorrhage: not reported
Welsch [9]	Pancreaticoduodenectomy with intraoperative coverage of the hepatic artery including the gastroduodenal artery stump using the pedicled falciform ligament wrap in a standardized fashion	Same surgical technique without wrapping of the regional vessels	90 days
Xu [13]	Gastroduodenal artery stump (GDA) was entirely wrapped by the teres hepatis ligamentum.	Same surgical technique without wrapping of the regional vessels	Not reported

### 3.2 Methodological evaluation of included studies

The methodological quality of included trials is summarized in Table 3. The Mantel-Haenszel random effects model was used to compute robustness and susceptibility to any outlier among these trials. The randomization of both randomised trials was done using R software package, and the concealment was done using opaque envelops [5,9]. Moreover, blinding was reported in one of the randomised trials [9]. The quality of the retrospective studies was analysed using the Scottish Intercollegiate Guidelines Network and Rangel et al. [23] and were found to be of fair quality [4,[10], [11], [12], [13]].

**Table 3:** Quality of the studies.

Study	Randomization technique	Concealment	Blinding	Intention to treat analysis	Ethical approval	SIGN score for retrospective studies
Matsuda [10]	Not applicable	Not applicable	Not applicable	Not reported	Not reported	Fair quality (score 9)
Meng [4]	Not applicable	Not applicable	Not applicable	No patient lost for follow up	Reported	Fair quality (score 13)
Mussle [5]	The randomization sequence using R software package	Via envelopes	Not reported	Reported	Reported	Not applicable
Okada [11]	Not applicable	Not applicable	Not applicable	Reported	Reported	Fair quality (score 12)
Tani [12]	Not applicable	Not applicable	Not applicable	Not reported	Not reported	Fair quality (score 10)
Welsch [9]	The randomization sequence using R software package	Via envelops	Yes	Reported	Reported	Fair quality (score 13)
Xu [13]	Not applicable	Not applicable	Not applicable	Not reported	Not reported	Fair quality (score)

### 3.3 The outcome of the primary variable

**Fig. 2:**
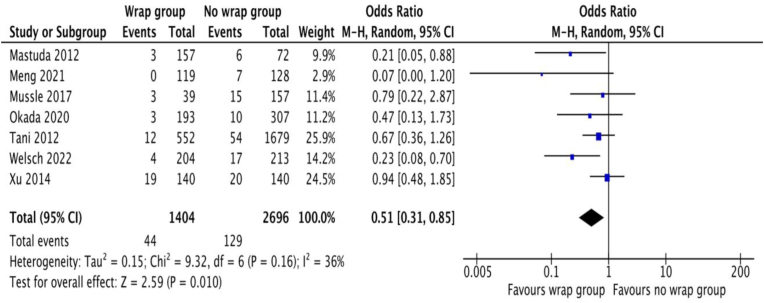
Forest plot showing the incidence of post operative extra-luminal haemorrhage after pancreaticoduodenectomy. The outcome is presented as odd ratio with 95% confidence interval.

The individual odds ratio (OR) and summated OR with (a) 95% confidence intervals for the random effects model meta-analysis of included studies are presented in Fig. 2. Therefore, in the random effects model analysis, the incidence of PPH was statistically lower in WG [odds ratio 0.51, 95%, CI (0.31, 0.85), Z = 2.59, P = 0.01]. There was moderate heterogeneity [Tau^2^ = 0.15; chi^2^ = 9.32; df = 6; I^2^ = 36%; p = 0.16] between the studies, however, statistically it was not significant. Despite combined analysis of 2 RCTs and 5 retrospective studies, moderate heterogeneity among included studies suggest reasonable acceptance to generate current evidence. Subgroup analysis of the RCTs and retrospective comparative studies separately also confirmed the positive role of regional vessels wrapping to reduce the risk of PPH.

## 4 Discussion

Current systematic review of two RCT and 5 retrospective studies on 4100 patients undergoing pancreaticoduodenectomy indicated that the wrapping of the regional vessels may be associated with significantly reduced risk of PPH resulting in reduced mortality and morbidity. The WG consisted of 1404 patients which is considered a good study sample considering the prevalence of pancreatic tumour suitable for surgical resection. Statistically, in the random effects model analysis, the incidence of extra-luminal haemorrhage was found to be lower in WG but moderate heterogeneity between the included studies was also encountered. The wrapping of regional vessels using any regional anatomical structure such as omentum, falciform ligament or ligamentum teres were all suitable in reducing the risk of PPH.

PPH is a life threating complication following pancreaticoduodenectomy; therefore several measures are taken during surgery in order to avoid this complication. The finding of this current meta-analysis of two RCTs and five retrospective studies on 4100 patients is consistent with the outcomes of a previously published meta-analysis [26]. There was significant diversity in the inclusion criteria, exclusion criteria and methodological assessment in the previous meta-analysis [26]. Due to the paucity of RCTs, the previous meta-analysis only analysed retrospective studies with fewer patients. To the best of our knowledge, this is the only meta-analysis of the highest number (4100) of patients (including 2 RCTs) reporting the effectiveness of regional vessel wrapping to reduce the risk of potentially life-threatening PPH. It provides relatively strong evidence to consider the routine use of wrapping of regional vessels in patients undergoing pancreaticoduodenectomy.

There are several limitations of this study. It is a combined analysis of RCTs and retrospective studies which is a potential source of biased evidence. The included RCTs are of reasonable quality but the retrospective comparative studies did not score well according to the criteria of quality assessment. A multicentre larger RCT is required to solidify the findings of this study before the standard recommendation of wrapping of the regional vessels following pancreaticoduodenectomy. Moreover, future studies ought to include a comparison of different types of wrapping material, in order to identify the optimal wrapping material and technique to reduce the incidence of PPH.

## Ethical approval

Not required.

## Sources of funding

None.

## Author contributions

Hussameldin M Nour: Idea conception, literature search, trial selection, data extraction, writing - original draft.

Dimitra Peristeri: literature search, trial selection, data extraction, review & editing.

Amiya Ahsan: literature search, trial selection, data extraction.

Shehram Shafique: literature search, trial selection, data extraction.

Prof Mansoor Khan: Writing - original draft, Writing - review & editing, Formal analysis. Muhammad S. Sajid: Data approval, data analysis, manuscript review and approval, supervision of the project.

## Registration of research studies

Registration unique ID: reviewregistry1432.

## Guarantor

Mr Muhammad S. Sajid.

## Consent

Not required.

## Provenance and peer review

Not commissioned, externally peer-reviewed.

## Declaration of competing interest

None.
